# Inner SPACE: 400-Micron Isotropic Resolution MRI of the Human Brain

**DOI:** 10.3389/fnana.2020.00009

**Published:** 2020-03-19

**Authors:** Timothy M. Shepherd, Michael J. Hoch, Mary Bruno, Arline Faustin, Antonios Papaioannou, Stephen E. Jones, Orrin Devinsky, Thomas Wisniewski

**Affiliations:** ^1^Department of Radiology, New York University, New York, NY, United States; ^2^Center for Advanced Imaging Innovation and Research (CAI2R), New York, NY, United States; ^3^Department of Radiology, University of Pennsylvania, Philadelphia, PA, United States; ^4^Department of Pathology, New York University, New York, NY, United States; ^5^Department of Radiology, Cleveland Clinic, Cleveland, OH, United States; ^6^Department of Neurology, New York University, New York, NY, United States; ^7^Department of Psychiatry, New York University, New York, NY, United States

**Keywords:** functional neurosurgery, atlas, MR microscopy, 3D visualization, teaching

## Abstract

**Objectives:**

Clinically relevant neuroanatomy is challenging to teach, learn and remember since many functionally important structures are visualized best using histology stains from serial 2D planar sections of the brain. In clinical patients, the locations of specific structures then must be inferred from spatial position and surface anatomy. A 3D MRI dataset of neuroanatomy has several advantages including simultaneous multi-planar visualization in the same brain, direct end-user manipulation of the data and image contrast identical to clinical MRI. We created 3D MRI datasets of the postmortem brain with high spatial and contrast resolution for simultaneous multi-planar visualization of complex neuroanatomy.

**Materials and Methods:**

Whole human brains (*N* = 6) were immersion-fixed in 4% formaldehyde for 4 weeks, then washed continuously in water for 48 h. The brains were imaged on a clinical 3-T MRI scanner with a 64-channel head and neck coil using a 3D T2-weighted sequence with 400-micron isotropic resolution (voxel = 0.064 mm^3^; time = 7 h). Besides resolution, this sequence has multiple adjustments to improve contrast compared to a clinical protocol, including 93% reduced turbo factor and 77% reduced effective echo time.

**Results:**

This MRI microscopy protocol provided excellent contrast resolution of small nuclei and internal myelinated pathways within the basal ganglia, thalamus, brainstem, and cerebellum. Contrast was sufficient to visualize the presence and variation of horizontal layers in the cerebral cortex. 3D isotropic resolution datasets facilitated simultaneous multi-planar visualization and efficient production of specific tailored oblique image orientations to improve understanding of complex neuroanatomy.

**Conclusion:**

We created an unlabeled high-resolution digital 3D MRI dataset of neuroanatomy as an online resource for readers to download, manipulate, annotate and use for clinical practice, research, and teaching that is complementary to traditional histology-based atlases. Digital MRI contrast is quantifiable, reproducible across brains and could help validate novel MRI strategies for *in vivo* structure visualization.

## Introduction

State-of-the-art clinical MRI provides excellent soft tissue contrast, yet does not resolve much of the complex, intricate internal anatomy of the human brain. For example, the thalamus appears relatively homogenous on conventional volumetric T1 images, yet contains many functionally distinct nuclei and projections from the spinal cord, brainstem, cortex, and adjacent basal ganglia (Carpenter et al., 1976). To teach and use neuroanatomy in research or clinical practice, we first learn the size, shape and relative positions of different structures using atlases based on serial single-plane histology stained sections of the human brain (Olszewski and Baxter, 1982; DeArmond et al., 1989; Warner, 2001; Mai et al., 2015; Haines, 2019). We then try to mentally juxtapose this knowledge onto multi-planar clinical MRI images to infer the location of functionally important structures based on the position in 3-dimensional brain space and relative to surface topography. For some structures the position relative to another MRI-visible structure may help (e.g., on susceptibility-weighted imaging, the subthalamic nucleus is lateral to the more visible red nuclei). Stereotactic atlases improve indirect structure localization (Schaltenbrand and Wahren, 1977), but do not account for individual variation and left-right asymmetries (Niemann and van Nieuwenhofen, 1999), or changes from aging and disease. This limits our ability to use MRI to recognize pathologic involvement of specific structures in individual clinical patients or quantify subtle changes during research investigations. Similarly, indirect anatomic targeting for functional neurosurgery limits our understanding of how this treatment actually works (Littlechild et al., 2003; Plaha et al., 2006). To help address this problem, several groups have provided labeled, high spatial resolution MRI datasets of individual brains using conventional MRI sequences and contrasts (Shen et al., 2012; Cho et al., 2015; Lusebrink et al., 2017).

An alternative is to create neuroanatomical atlases using MRI microscopy of human brain samples – a common microscopy paradigm is to dissect a specific brain structure, like the brainstem, then image at spatial MRI resolutions not possible *in vivo* using a smaller coil and narrow-bore high-field research magnet (Fatterpekar et al., 2002, 2003; Aggarwal et al., 2013). These are helpful for research, teaching and sometimes help infer a structure’s location in patients, but remain removed from clinical practice – e.g., advanced diffusion tractography derived contrast maps of an isolated structure are not intuitive to radiologists or other clinicians. Instead, the whole postmortem brain can be imaged with common clinical MRI contrast mechanisms that appear similar to myelin-stained histology (Naidich et al., 2009) – recent reports used 2D turbo spin echo (TSE) with anisotropic 0.098 mm^3^ voxels (Hoch et al., 2019a, b). A 2D approach has limitations including instrument restrictions to reducing image slice thickness and the need for *a priori* prescription of imaging planes. Similar to histology atlases, these prior whole brain MRI studies present labeled serial images in multiple planes, but the interested reader is unable to manipulate the 3D data directly themselves in multiple planes simultaneously to investigate the location and spatial relationships for individual structures of interest. We now demonstrate using a 3D T2-weighted sequence to characterize the human brain with 400-μm isotropic resolution (0.064 mm^3^ isotropic voxels). The 3D T2-weighted sequence has 3 key advantages over previous work; (1) reduced partial volume effects to improve both spatial and contrast resolution of sub-millimeter internal brain structures (2) the ability to repeatedly generate multiplanar and/or anatomic-specific oblique reformats from the same dataset, and (3) generation of an online 3D dataset as a resource that can be distributed to interested readers for local user-directed exploration, investigation, and teaching.

## Materials and Methods

### Sample Procurement and Preparation

Anonymized whole brain samples were obtained from an institutional-review board approved study from the local Alzheimer’s Disease Research Center. This protocol utilized *ex vivo* MRI screening prior to gross pathologic assessment, brain cutting and histopathology for research investigation. For each subject, the brain was removed intact by the local medical examiner. In some samples, small frontal and occipital lobe blocks were removed via coronal cuts then frozen immediately for immunohistochemistry. The remaining intact brain was suspended in a 4% formaldehyde solution for 4–6 weeks, then washed with water for 48 h to eliminate MRI relaxation changes from aqueous aldehyde fixative (Dawe et al., 2009; Shepherd et al., 2009b). Adult brains imaged for this report (*N* = 6) had no gross pathological abnormality identified by a board-certified neuroradiologist and neuropathologist, no premorbid clinical diagnosis of cognitive impairment and pre-refrigeration postmortem interval < 24 h. Brain-cutting and histological assessment of structures performed after MRI microscopy followed standard accepted neuropathology clinical protocols – no histopathologic abnormalities were present in the 6 brains used for this study.

### Whole Brain MR Microscopy

Each brain was immersed under water inside a 3D-printed container conforming to a 64-channel head and neck coil on a 3-T Prisma MRI scanner (Siemens Healthcare, Erlangen, Germany). Plastic spacers were wedged between the container and brain to prevent motion. Scout sequences identified brain position, then a 3D SPACE (“Sampling Perfection with Application optimized Contrasts using different flip angle Evolution”) sequence was obtained. Preliminary studies explored different effective echo time, relaxation time, turbo factor, signal averaging, the use of partial Fourier acquisitions and variable flip angles within the SPACE sequence to generate optimal contrast as determined by consensus between two neuroradiologists. Final SPACE parameters are contrasted to the clinical standard on the same MRI scanner in Table 1. A complete version of the protocol will be provided by the authors to interested readers. In this report, we use the more general acronym TSE to describe the T2-weighted contrast generated (but consider SPACE or “fast spin echo” equivalent). The 400-μm isotropic resolution imaging dataset was reconstructed in coronal, sagittal and axial planes relative to the anterior-posterior commissural plane. Additional images were derived in oblique planes to illustrate the utility of the 3D data to demonstrate specific anatomic relationships of the brain. The original 400-micron isotropic dataset also was used at a separate institution and co-registered to selected clinical cases to improve lesion anatomic localization that was unclear on the original clinical MRI studies. Rigid affine registration between the cadaver brain and the patient images was manually performed by board-certified neuroradiologist sequentially in the three 2D planes based on a field-of-view restricted to the brainstem using clinical DICOM viewer (IMPAX 6.3, Agfa, Mortsel, Belgium) until there was convergence of co-registration as confirmed by correspondence between major visible anatomic landmarks.

**TABLE 1:** Comparison of clinical and microscopy 3D T2 SPACE sequence parameters* using clinical 3-T MRI system with 64-channel head and neck coil.

**Application**	**Clinical protocol**	**Microscopy protocol**
Repetition time	3200 ms	2500 ms
Echo time	422 ms	25 ms
Effective echo time	111 ms	25 ms
Turbo factor	257	17
Echo spacing	6.49 ms	3.49 ms
Echo train duration	862 ms	110 ms
Bandwidth/pixel	751 Hz	337 Hz
Flip angle mode	Variable	Constant (140^*o*^)^#^
Slices per slab	176	320
GRAPPA acceleration factor	2	2
Field-of-view	256 mm	205 mm
k-space ordering	Linear	Radial
Slice partial Fourier	6/8	Off
Base resolution	256	512
Isotropic voxels	1-mm	0.4-mm
Averages	1	2
Time	3 min 33 s	7 h 28 s

*Full sequence parameters for both protocols available upon request. ^#^A key defining feature of the SPACE sequence is variable flip angle, which was not necessary for short echo trains, but the manufacturer SPACE sequence architecture was still used.

## Results

The 400-micron isotropic resolution 3D SPACE sequence provided excellent contrast resolution of the cortical layers,

deep gray nuclei, brainstem and cerebellar structures. An Online movie with axial images reconstructed parallel to the intercommissural plane of the entire postmortem brain is provided to illustrate the quality and contrast resolution generated with the 3D SPACE sequence. In general, the TSE contrast emphasizes mobile proton density or T2 differences which appear similar to myelin-based stains (e.g., Weigert) published in histology-derived brain atlases (Olszewski and Baxter, 1982; DeArmond et al., 1989; Warner, 2001; Haines, 2019). Densely myelinated white matter structures (callosum, cerebral and cerebellar peduncles) appear dark, while cortex and deep gray nuclei appear bright (Haines, 2019). Many smaller brainstem and diencephalon pathways, as well as internal features of certain deep gray nuclei (e.g., globus pallidus) include intermediate gradations of TSE signal intensity. This reflects more complex tissue mesoscopic structure that includes varying amounts of intravoxel myelin.

**FIGURE 1:**
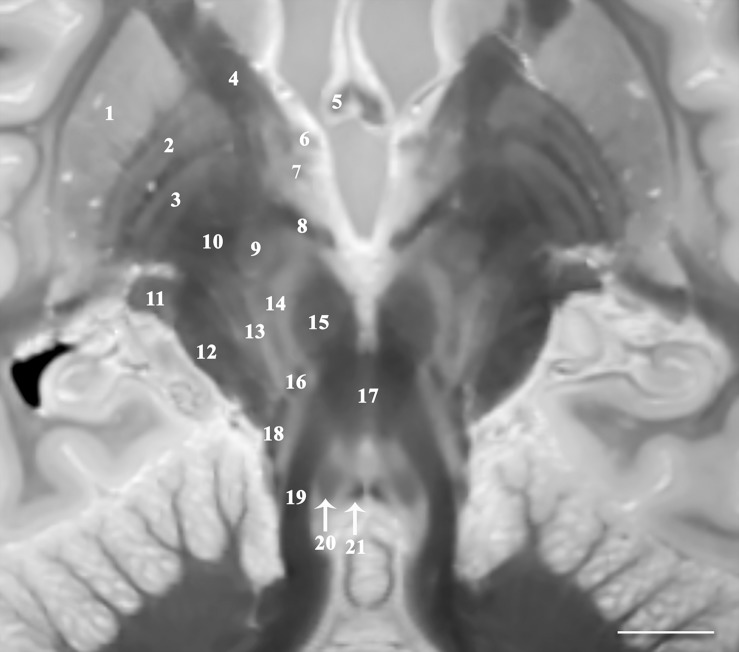
Image parallel to the superior cerebellar peduncles and their decussation that is 33^*o*^ anterosuperior-to-posteroinferior oblique to the coronal intercommissural plane demonstrates relative spatial relationships of the midbrain, subthalamic and basal ganglia structures including putamen (1), globus pallidus externa (2) and interna (3), internal capsule (4), fornix (5), ansa peduncularis (6), ansa lenticularis (7), lenticular fasciculus (8), subthalamic nucleus (9) and fasciculus (10), lateral geniculate nucleus (11), cerebral peduncle (12), pallidonigral/corticonigral/nigrostriatal tracts (13), substantia nigra (14), red nucleus (15), medial leminiscus (16), decussation of superior cerebellar peduncles (17), spinothalamic tract (18), superior cerebellar peduncles (19), central tegmental tract (20), and medial longitudinal fasciculus (21) [scale bar = 10 mm]. Note multiple internal lamina of the globus pallidus.

**FIGURE 2:**
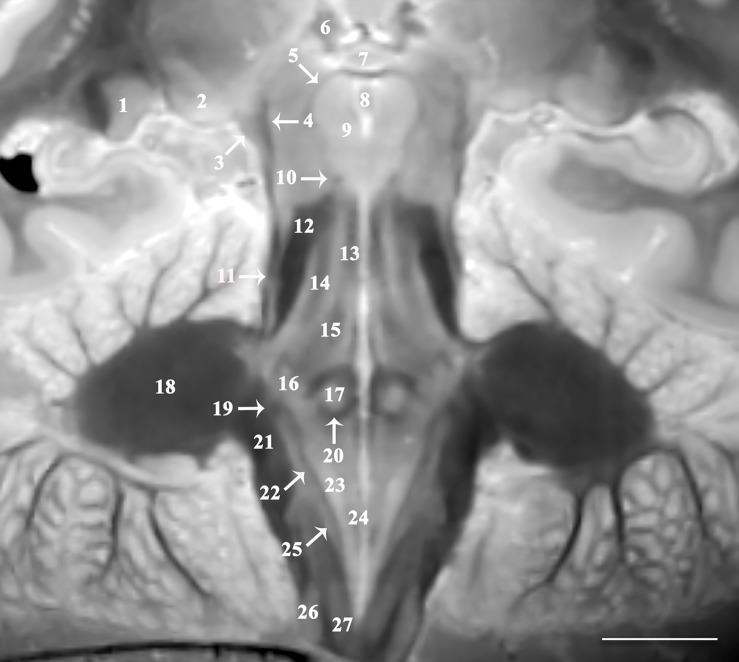
Image oriented parallel and deep to the rhomboid fossa that is 15^*o*^ anterosuperior-to-posteroinferior oblique to the coronal intercommissural plane provides excellent visualization of lateral (1) and medial geniculate nuclei (2), brachium of the inferior colliculus (3), spinothalamic tract (4), mesencephalic trigeminal nucleus (5), habenular nucleus (6), posterior commissure (7), cerebral aqueduct (8), oculomotor nucleus (9), trochlear nerve (10), lateral lemniscus (11), superior cerebellar peduncle (12), medial longitudinal fasciculus (13), central tegmental tract (14), mesencephalic trigeminal tract (15), trigeminal nucleus (16), abducens nucleus (17), middle cerebellar peduncle (18), vestibular division of the vestibulocochlear nerve (19), genu of facial nerve (20), inferior cerebellar peduncle (21), solitary tract (22), dorsal motor vagal nucleus (23), hypoglossal nucleus (24), solitary nucleus (25), cuneate (26), and gracile nucleus (27) [scale bar = 10 mm]. Note the proximal optic radiations forming the dark lateral border of the lateral geniculate nucleus. The obliquity of the coronal image through both the spinothalamic tract (4) and the lateral lemniscus (11) gives the appearance of one contiguous structure. These are easily resolved as two distinct structures on the complete downloadable 3D dataset.

**FIGURE 3:**
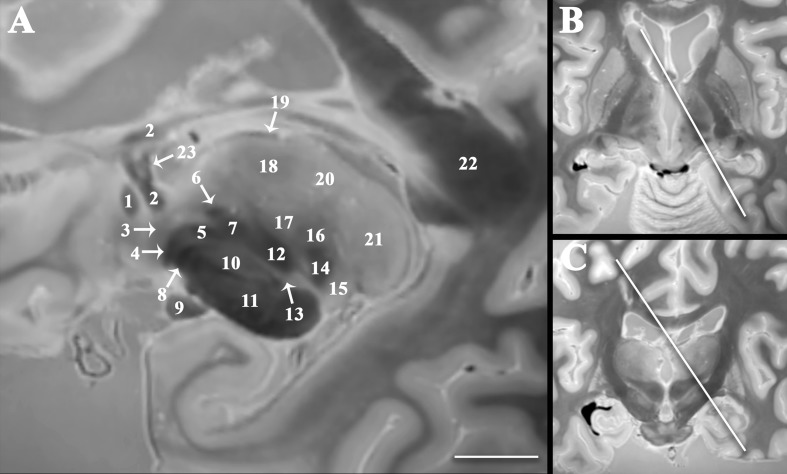
Double oblique sagittal image oriented orthogonal to the thalamic termination of the dentatorubrothalamic tract **(A)** to improve visualization of the spatial relationships between the subthalamic region, cerebellothalamic, and pallidothalamic pathways important to emerging functional neurosurgery treatments for movement disorders. Panel **(B)** demonstrates angulation in the axial plane 35^*o*^ relative to the sagittal plane (anteromedial-to-posterolateral). Panel **(C)** demonstrates angulation in the coronal plane 30^*o*^ relative to the sagittal plane (superomedial-to-inferolateral). Labeled structures include anterior commissure (1), fornix (2), ansa peduncularis (3), ansa lenticularis (4), lenticular fasciculus (5), thalamic fasciculus (6), H field of Forel (7), pallidonigral tract (8), optic tract (9), subthalamic nucleus (10), cerebral peduncle (11), dentatorubrothalamic tract (12), zona incerta (13), medial lemniscus (14), medial geniculate nucleus (15), ventral posterior nucleus (16), ventral intermediate nucleus (17), ventral lateral nucleus (18), stria medullaris (19), dorsomedial nucleus (20), pulvinar (21), splenium of corpus callosum (22), and direct fornix projection to the anterior thalamic nucleus (23) [scale bar = 10 mm].

**FIGURE 4:**
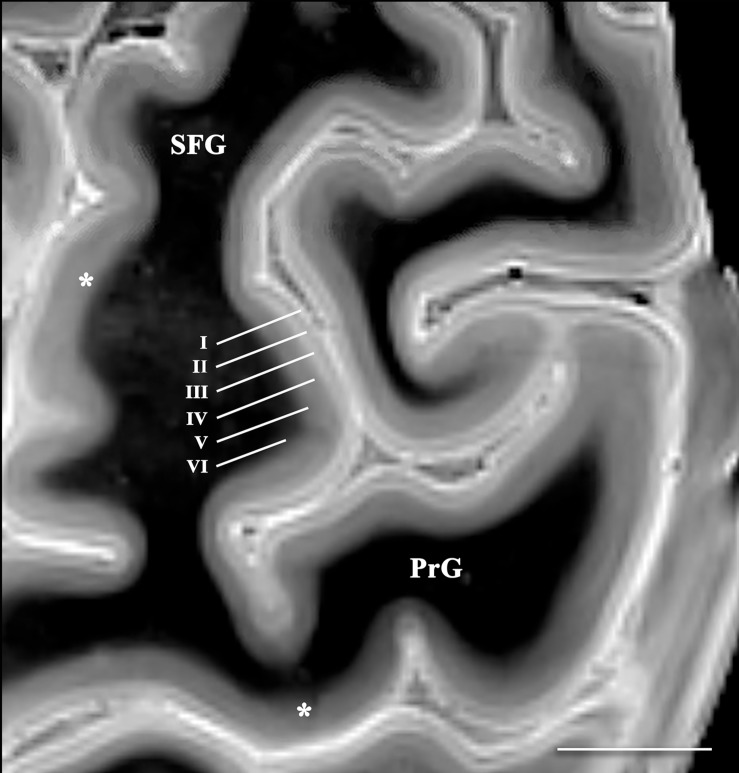
Axial image of the frontal cerebral cortex near the superior convexity of the left hemisphere (PrG, precentral gyrus; SFG, superior frontal gyrus) [scale bar = 10 mm]. T2 SPACE images with 400-micron isotropic voxels provide adequate spatial and contrast resolution to visualize horizontal cortical layers (annotated along the lateral bank of the superior frontal gyrus). The layers vary in signal intensity and thickness. Contrast discrimination of the deeper layers (particularly layer V) is diminished in the primary and supplementary motor cortices (*).

**FIGURE 5:**
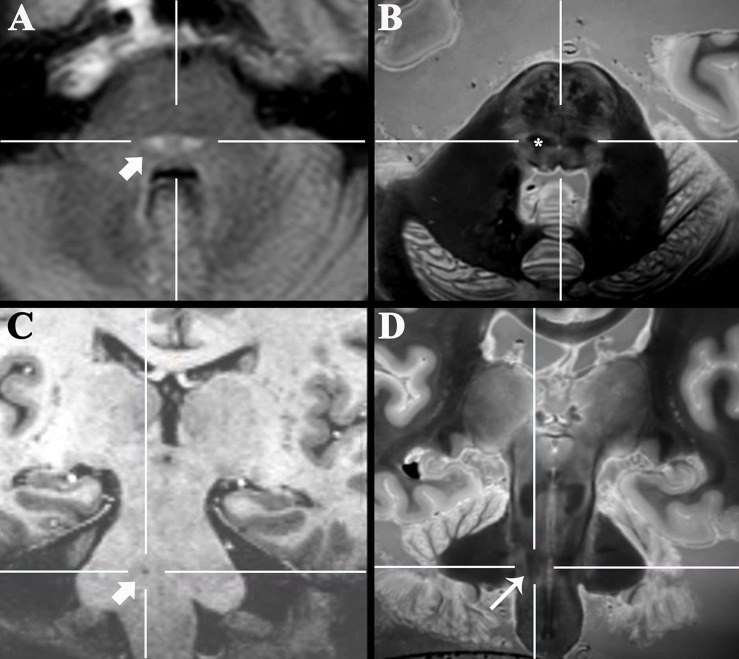
Axial 3-T FLAIR clinical image of a 47-year-old patient with new sensory loss and suspected Multiple Sclerosis **(A)** demonstrated new bilateral FLAIR-bright demyelinating lesions in the pons (arrow). Coregistered 3D SPACE *ex vivo* image **(B)** suggested the lesions were in the medial lemniscus (*, labeled unilaterally). Oblique coronal 7-T 3D T1-weighted image **(C)** of a 77-year-old patient that presented with progressive dysarthria, palatal myoclonus and hypertrophic olivary degeneration (not shown). The precise anatomic location of a tiny T1-dark lacunar infarct in the right pons (arrow) could not be determined even with 7-T MRI. Coregistered 3D SPACE *ex vivo* image at 3-T **(D)** suggests the lesion is in the central tegmental tract (long arrow).

The 3D data can be visualized in multiple planes simultaneously to better localize specific structures, or to generate oblique image orientations that illustrate specific neuroanatomical relationships. We have provided 3 examples from the same dataset to emphasize this capability, but note that the contrast is highly reproducible across individual brains. Figure 1 illustrates an oblique axial-coronal reconstruction parallel to the superior cerebellar peduncles and their decussation. Oblique images help illustrate this complex transition between the midbrain and diencephalic structures which can be confusing due to its ∼90^*o*^ kyphotic angulation in bipedal humans compared to other mammals. Figure 2 illustrates an oblique axial-coronal reconstruction parallel and just deep to the rhomboid fossa. Figure 3 re-orients a sagittal image obliquely relative to both the coronal and axial planes to illustrate the nuclei, pallidothalamic and cerebellothalamic pathways in the subthalamic region relevant to functional neurosurgery targeting in movement disorders. Figure 4 demonstrates TSE image contrast resolution of cortical layers, which appear to vary in thickness and signal intensity. Figure 5 shows the potential of the microscopy dataset to enhance clinical practice through two selected cases of lesion anatomic co-localization. A systematic annotated atlas of neuroanatomy is beyond the scope of this brief report, but the reader is encouraged to explore the data further. The original source data for 6 individual brains can be provided to interested readers (each file size ∼1GB).

## Discussion

We have produced unlabeled high-resolution digital 3D MRI datasets of neuroanatomy as an online resource for readers to download, manipulate, annotate and use for teaching that is complementary to traditional histology-based atlases. The TSE MRI image contrast recapitulates histology stains of myelin supporting the anatomic assignments in the current figures. Compared to recent publications using 2D TSE of the brainstem and basal forebrain anatomy (Hoch et al., 2019a, b) the 3D TSE produces 35% smaller image voxels with improved contrast resolution of small structures. This 3D MRI atlas also has advantages over histology-based brain atlases. Neuroanatomy is taught using histology-stained sections, but the majority of practicing clinicians never encounter brain histology again in practice. MRI images are inherently digital facilitating online dissemination, end-user manipulation of contrast and quantification. Unlike 2D histology atlases (Olszewski and Baxter, 1982; DeArmond et al., 1989; Warner, 2001; Haines, 2019) or previous 2D MRI microscopy (Hoch et al., 2019a, b), a single 3D dataset can be used to view the same structure in the same individual brain using multiple planes simultaneously. Those image plane orientations can be prescribed by the individual user *post hoc* and this process can be repeated in the same dataset for different orientations and structures. The interested reader can manipulate the images themselves to facilitate individual learning. This collection should help clinicians understand complex spatial relationships for regions that are important to emerging functional neurosurgery applications (e.g., subthalamic region in Figure 3).

While a 7-h scan is prohibitively long relative to current clinical MRI of living patients, the required resources for MRI atlas image creation are small compared to conventional atlases currently used for teaching neuroanatomy. Serial planar sections of 3 individual human brains using axial, coronal and sagittal planes, respectively, with conventional histology-staining would require a year from a full-time technician and $30,000 in supplies. Sectioning and staining whole brain slices requires special equipment and technical expertise that are not widely available. There is a real risk that brain section orientation will be wrong, or that portions of the sample will be damaged before whole brain preparation is complete. Hence, the 10th edition of a modern atlas for teaching neuroanatomy still uses brain sections published with the 1st edition in 1983 (Haines, 2019). A 3D MRI dataset of an autopsy brain can be generated using a clinical 3-T MRI scanner overnight – this also makes it much more feasible to generate 3D datasets from many individual brains. A recent report described creation of a 3D 100-micron isotropic resolution MRI dataset using a 100-hr scan (∼4 days) on a 7-T MRI system – the technically challenging and long acquisition may limit the ability to generate a library of multiple individual brains using such a protocol (Edlow et al., 2019).

Understanding the basis for 3D TSE contrast observed here will be important for selecting appropriate compromises if this sequence is to be adapted in the future for *in vivo* application – this will be particularly challenging because of subject motion and limits to scan time tolerated by living subjects. Contrast generation with the 3D TSE sequence represents a complex interaction of sequence parameters (Mugler, 2014). Smaller isotropic voxels may be the dominant source of excellent contrast resolution of neuroanatomic structures in 3D TSE images, but there also may be contributions from additional 3D TSE sequence changes afforded by longer scan times (Table 1) and surreptitious, but advantageous MRI relaxation changes in the postmortem, formaldehyde-fixed brain. Reduced echo spacing and turbo factor decreased T2 blurring across k-space thereby increasing image sharpness. Avoiding partial Fourier acquisitions also reduced image blurring. Radial k-space ordering enables shorter effective echo time. Reduced turbo factor created more precise effective TE (and T2-weighting). Reduced bandwidth increased signal-to-noise. The short echo train makes variable flip angle (VFA) unnecessary – in clinical 3D SPACE VFA is used to reduce contributions from stored magnetization, T1 relaxation and stimulated echoes associated with *long echo trains*. Higher isotropic spatial resolutions of the whole postmortem brain may be possible, however, this will require changing MRI manufacturer sequence parameter limitations (created for patient safety) and increasing MRI scanner working memory capacity prior to image reconstruction.

The current study has limitations. TSE image contrast and *ex vivo* brain relaxation parameters may be altered compared to the *in vivo* brain by agonal pathophysiology (Dos Santos et al., 2014); postmortem interval (Shepherd et al., 2009a); procurement-associated brain distortion, cuts or intraventricular air; formaldehyde fixation (Shepherd et al., 2009b); and imaging at room temperature (Ruder et al., 2012). We empirically observed that relaxation time above 2 s only increased scan duration (an undesirable result), but did not visibly affect contrast via T1 relaxation mechanisms. Further, the short effective echo time and turbo factor should minimize the impact of formaldehyde fixation, postmortem interval and temperature induced differences to tissue T2 or bound water fraction (i.e., magnetization transfer effects). All *ex vivo* datasets were acquired on a clinical 3-T scanner using a modified SPACE sequence. Potential improvement of *ex vivo* structure assignments may result from a combined approach utilizing different contrasts such as diffusion, susceptibility or higher field strengths (7-T) (Edlow et al., 2019). Ultra-high field scanners can be prone to increased geometric distortion and signal loss at the skull base but have potential to improve resolution for *in vivo* acquisitions (Zeineh et al., 2014).

There are several compelling future directions for this work. Modifying this MRI sequence to be feasible in living human subjects requires more systematic investigation of the dominant contributing factors to contrast resolution. A convolutional neural network could be used to de-noise data from *in vivo* acquisitions when scan time is more limited (Zhang et al., 2017). Detailed anatomic assignment of all visualized structures with histological coregistration in the same brain is beyond the scope of this report. The 3D TSE sequence described here should be applicable to living primates, particularly using higher field magnets, smaller coils, and prolonged anesthesia – creating the potential for *in vivo* validation and structure-function studies. We demonstrated that individual 3D TSE datasets can be co-registered with clinical images from individual patients to estimate specific structure locations, but an atlas and template derived from a population of brains would be more accurate. A template derived from multiple brains would reduce inherent individual variability and may be useful in guiding functional surgery. This protocol also could help validate advanced MRI applications for visualizing specific structures (e.g., diffusion tractography). We plan to collect more 3D MRI datasets from normal and diseased human brains to create a free online resource for neuroanatomy education and research.

## Data Availability Statement

The raw data supporting the conclusions of this article will be made available by the authors, without undue reservation, to any qualified researcher.

## Ethics Statement

The studies involving human participants were reviewed and approved by the New York University Institutional Review Board.

## Author Contributions

All authors contributed to the study design, data collection, analysis, and interpretation, helped to prepare and revise the manuscript, approved the submission for publication, and agreed to be accountable for all aspects of this work.

## Conflict of Interest

The authors declare that the research was conducted in the absence of any commercial or financial relationships that could be construed as a potential conflict of interest.

## Supplementary Material

VIDEO S1 Axial images of a whole postmortem brain imaged at 400-micron isotropic resolution using T2 SPACE MRI sequence. A manually drawn mask was applied to eliminate background signal from water and plastic spacers used to secure the brain. The original 3D DICOM datasets for 6 anonymized individual brains are provided online for distribution to interested readers – the data can be uploaded into a clinical PACS or freeware image analysis tools for simultaneous multiplanar visualization of specific structures of interest to individual readers.

## Abbreviations

PrG: precentral gyrus
SFG: superior frontal gyrus
SPACE: sampling perfection with application optimized contrasts using different flip angle evolution
TSE: turbo spin echo
VFA: variable flip angle.
